# Epilepsy in Paediatric Palliative Care: Prevalence, Clinical Correlations and the Development of a Consensus-Based Seizure Management Protocol

**DOI:** 10.3390/medicina62061150

**Published:** 2026-06-12

**Authors:** Mihaela Hizanu Dumitrache, Camer Salim, Alina Plesea-Condratovici, Dana Elena Mîndru, Mădălina Duceac Covrig, Eva Maria Elkan, Carmen Laura Cristescu Budala, Irina Luciana Gurzu, Petruța Iuliana Moraru, Mirela Mătăsaru, Mădălina Nicoleta Matei, Letiția Doina Duceac

**Affiliations:** 1 Doctoral School of Biomedical Sciences, Faculty of Medicine and Pharmacy, “Dunarea de Jos” University of Galați, 47 Domnească Street, 800008 Galați, Romania; mh202@student.ugal.ro (M.H.D.); madalinaduceac@yahoo.ro (M.D.C.); matasarumirela@gmail.com (M.M.); 2 Department of Emergency Medicine, Faculty of Medicine, “Ovidius” University of Constanta, 900470 Constanța, Romania; salimcamer@yahoo.com; 3 Research Centre in the Medical-Pharmaceutical Field, Faculty of Medicine and Pharmacy, “Dunărea de Jos” University of Galați, 47 Domnească Street, 800008 Galați, Romania; cojocarumariaeva@yahoo.com (E.M.E.); laura.cristescubudala@gmail.com (C.L.C.B.); iuliana.moraru@ugal.ro (P.I.M.); madalina.matei@ugal.ro (M.N.M.); letimedr@yahoo.com (L.D.D.); 4 Department of Pediatrics, Faculty of Medicine, Grigore T. Popa University of Medicine and Pharmacy Iasi, 16 Universitatii Street, 700115 Iași, Romania; 5 Department of Preventive Medicine and Interdisciplinarity, Faculty of Medicine, Grigore T. Popa University of Medicine and Pharmacy Iasi, 16 Universitatii Street, 700115 Iași, Romania; irina-luciana.gurzu@umfiasi.ro

**Keywords:** paediatric palliative care, epilepsy, seizures, life-limiting illnesses, children

## Abstract

*Background and Objectives*: Seizures and epilepsy are common in children with life-limiting illnesses, particularly in the context of severe neurological impairment. However, data on the clinical profile of these patients in paediatric palliative care are limited, and the lack of standardised protocols adapted to this context represents a clinical challenge. To assess the prevalence of epilepsy and associated clinical characteristics in children receiving paediatric palliative care and to develop a clinical protocol for the management of seizures based on expert consensus. *Materials and Methods*: A retrospective observational study was conducted, based on the analysis of clinical data from children registered in a paediatric palliative care service (Lumina Association, Romania). Demographic, diagnostic, neurological status and anticonvulsant treatment data were collected. Based on the results obtained and the existing literature, a clinical protocol was developed using a modified Delphi method approach, involving a multidisciplinary panel of experts and two rounds of evaluation of clinical statements. *Results*: A total of 101 patients (54.5% boys and 45.5% girls) were included, with a mean age of 7.2 ± 4.7 years. Epilepsy was documented in 32.7% of patients and was significantly associated with a neurological diagnosis (*p* = 0.008), severe neurodevelopmental delay (*p* = 0.032) and severe motor impairment (*p* = 0.036). The Delphi process led to the validation of 13 clinical recommendations, the majority of which achieved a strong level of consensus (>85%). *Conclusions*: Epilepsy is common and closely associated with severe neurological impairment in paediatric palliative care. The integration of systematic neurological assessment and the implementation of a consensus-based clinical protocol can support a more structured approach to seizure management in paediatric palliative care.

## 1. Introduction

Paediatric palliative care is a complex field of medicine, aimed at improving the quality of life of children diagnosed with life-limiting or life-threatening illnesses, through a holistic approach that integrates symptom control, psychosocial support and interdisciplinary interventions [1,2]. Unlike the palliative model applied to adults, paediatric care involves specific considerations related to neurocognitive development, dependence on the family and the aetiological heterogeneity of diseases, which necessitates the continuous adaptation of therapeutic strategies [3].

In this context, neurological symptoms play a central role, being reported in a significant proportion of paediatric patients receiving palliative care. Seizures, in particular, represent one of the most common and distressing clinical manifestations, having direct implications for the patient’s comfort, as well as for the stress experienced by the family and carers [4,5]. Seizure episodes can be unpredictable, recurrent and difficult to control, contributing to further deterioration in functional status and increasing the complexity of care. The frequency of seizures and the need for continuous monitoring can significantly increase the psycho-emotional burden on carers, particularly in the context of home care for children with severe neurological impairment [6].

Epilepsy is common among children with severe neurological conditions, including structural encephalopathies, rare genetic disorders and metabolic disorders, and is often associated with severe neurodevelopmental delay and major motor disability [7,8]. The complexity of caring for these patients is compounded by the frequent association of neurological disorders with multiple chronic comorbidities that can influence both treatment adherence and tolerance to repetitive medical interventions [9]. In these populations, epilepsy is often treatment-resistant, requiring complex therapeutic regimens and continuous monitoring. Furthermore, the interaction between the progression of the underlying disease and inadequate seizure control contributes to a significant clinical burden.

Severe forms of epilepsy in children, particularly those associated with developmental and epileptic encephalopathies, structural brain abnormalities or genetic disorders, frequently follow a progressive clinical course characterised by recurrent seizures, pharmacoresistance and worsening neurocognitive impairment [10,11,12]. Persistent epileptic activity may contribute to further deterioration of motor, cognitive and behavioural functions, negatively affecting functional autonomy and quality of life [12]. Children with severe neurological impairment and refractory epilepsy often require repeated hospitalisations and complex supportive interventions, including enteral feeding and respiratory support [11,13]. In the context of paediatric palliative care, understanding the natural evolution of severe epilepsy is essential for establishing realistic therapeutic goals focused on symptom control, comfort and family-centred care [13,14].

The management of seizures within paediatric palliative care presents specific challenges. Infrastructure limitations, reduced access to specialist services, and a shortage of staff trained in paediatric palliative care can negatively impact the continuity of neurological monitoring and rapid intervention in the event of acute seizure episodes [15].

The administration of anticonvulsant treatment may be hampered by dysphagia, altered neurological status or difficult venous access, and the need for rapid intervention frequently arises outside the hospital setting, in the home context [16]. In this regard, the use of alternative routes of administration (oral, intranasal, rectal) and the development of contingency plans become essential for preventing severe complications, including status epilepticus [17].

Although the international literature highlights the importance of seizure control in paediatric palliative care, epidemiological data on the prevalence of epilepsy and associated clinical characteristics in this population remain limited, particularly in Eastern Europe [18]. The lack of standardised registries and the variability in care models contribute to this knowledge gap, highlighting the need for observational studies to describe the clinical profile of these patients in real-world practice settings.

In Romania, the distribution of paediatric palliative care services remains uneven, and the high prevalence of severe neurological conditions among children with life-limiting illnesses highlights the need to develop integrated strategies for neurological and symptomatic management [19].

In the absence of standardised guidelines tailored to the specific needs of paediatric palliative care, the development of clinical recommendations is frequently based on integrating available evidence with expert experience. In this context, the Delphi method is a validated approach for reaching consensus in fields characterised by limited or heterogeneous evidence, and is widely used in the development of clinical guidelines and practice protocols [20,21]. Modified Delphi approaches, which use predefined items and a reduced number of evaluation rounds, are considered particularly useful when there is already a preliminary evidence base and the aim is to validate and operationalise this into clinically applicable recommendations [22,23].

In the field of paediatric palliative care, where case complexity and variability in care settings are high, such methods can facilitate the development of pragmatic tools for symptom management, including seizures.

Understanding the distribution of epilepsy and associated factors among children in palliative care is essential for developing tailored therapeutic strategies, optimising symptom management, and improving the quality of life for patients and their families.

In this context, the study aims to assess the prevalence of epilepsy and associated clinical characteristics in children receiving paediatric palliative care and to develop a clinical protocol for seizure management based on expert consensus.

## 2. Materials and Methods

### 2.1. Study Design

A retrospective observational study was conducted, based on the analysis of clinical data collected from the current practice of a specialist paediatric palliative care service. The retrospective design was chosen to allow the assessment of clinical characteristics and the prevalence of epilepsy in a real-world cohort of patients with life-limiting illnesses, in line with methodologies frequently used in palliative care research [24,25].

This type of design is appropriate in contexts where experimental interventions are unethical or unfeasible, and the primary objective is to describe and analyse the relationships between existing clinical variables.

The study was conducted within a paediatric palliative care service in Romania, namely the Lumina Association, a non-governmental provider offering integrated services (palliative care, inpatient care, home-based palliative care, medical consultations, psychological and social support).

The service caters to patients from diverse socio-economic backgrounds, including a significant proportion from rural areas, a factor relevant to access to care and continuity of treatment. The care model is family-centred and includes interdisciplinary interventions, in line with international recommendations in the field of paediatric palliative care [26].

### 2.2. Participants

The study population included children registered with the paediatric palliative care service during the period analysed (2025), according to data available in the clinical database.

Inclusion criteria: age < 18 years; confirmed diagnosis of a life-limiting or life-threatening illness, in accordance with internationally accepted definitions [27]; active registration with the paediatric palliative care service; availability of relevant clinical data in the clinical observation form—palliative care.

Exclusion criteria: incomplete medical records or lack of essential information regarding neurological status or treatment; patients in whom the presence or absence of epilepsy could not be confirmed.

Participant selection was carried out consecutively to reduce the risk of selection bias and to accurately reflect real-world clinical practice.

### 2.3. Data Collection

Data were retrospectively extracted from clinical observation forms and associated medical documents (medical letters, assessments and interdisciplinary plans).

The information was entered into a structured, anonymous electronic database created specifically for this study, with a view to standardising variables and reducing transcription errors. The retrospective standardisation of clinical variables aimed to reduce data heterogeneity and increase the reproducibility of the analysis in a paediatric population characterised by high diagnostic complexity.

The following categories of data were collected: demographic data: age, sex, place of origin (urban/rural); clinical data: primary diagnosis, disease category (neurological, oncological, genetic/metabolic, etc.); neurological status: presence of structural brain damage, level of neurocognitive development, severity of motor deficit; epilepsy: diagnosis documented in the medical record, based on clinical assessment and/or neurological investigations; anticonvulsant treatment: type of medication, number of drugs used and any difficulties with administration.

Additional clinical information was collected from available medical records whenever documented, including neuroimaging findings, electroencephalographic (EEG) evaluations, feeding support requirements, respiratory support and associated chronic comorbidities. Due to the retrospective nature of the study and the heterogeneity of clinical documentation, EEG investigations had not been performed systematically in all patients and were not available in a standardised format suitable for quantitative analysis. When available, EEG reports described background slowing, multifocal epileptiform discharges or diffuse encephalopathic abnormalities, findings frequently associated with severe neurological impairment and developmental epileptic encephalopathies [10,11,28]. Laboratory investigations were inconsistently documented and, therefore, were not included in the comparative statistical analysis. These additional clinical variables were reviewed descriptively in order to provide a broader characterisation of the study population and its neurological complexity [5,13,14].

Epilepsy was defined in accordance with the criteria of the International League Against Epilepsy (ILAE), namely the presence of at least two unprovoked seizures or the need for long-term antiepileptic treatment [29].

### 2.4. Variables Analysed

Primary variable: presence of epilepsy (binary variable: yes/no).

Secondary variables: category of underlying disease; presence of structural brain damage (confirmed by imaging or clinically); neurodevelopmental delay (classified as severe based on clinical assessments); motor impairment (clinically assessed and classified as severe when significantly limiting the patient’s autonomy); need for medical support (e.g., enteral nutrition, respiratory devices), as an indicator of clinical complexity.

The selection of these variables was guided by the specialist literature, which highlights the correlation between epilepsy and the severity of neurological impairment in the paediatric population [5,7].

Statistical analysis was performed using standard descriptive and inferential methods with IBM SPSS Statistics, version 26.0 (IBM Corp., Armonk, NY, USA). Continuous variables were expressed as mean ± standard deviation (SD), following verification of their distribution. Categorical variables were presented as absolute frequencies and percentages.

The Chi-square (χ^2^) test, suitable for analysing differences between categorical variables, was used to compare the groups (patients with epilepsy vs. without epilepsy).

The level of statistical significance was set at *p* < 0.05, in accordance with the standards used in observational clinical studies [30].

The analysis was exploratory in nature, given the sample size and the descriptive nature of the study.

### 2.5. Ethical Considerations

The study was conducted in accordance with the Declaration of Helsinki and was approved by the Ethics Committee of the Lumina Association (opinion no. 2 of 10 April 2026) [31].

Data were anonymised prior to analysis, and access to patient information was restricted exclusively to the research team. Given the retrospective nature of the study and the use of existing data, individual informed consent was deemed inapplicable, in accordance with local regulations and accepted practices in observational studies [32].

### 2.6. Development of a Consensus-Based Clinical Protocol

Based on the results of the present observational study, a clinical protocol for the management of seizures in paediatric palliative care was developed using a modified Delphi method approach.

The use of the Delphi method was justified by the absence of standardised guidelines specific to the paediatric palliative care context and by the variability in clinical practice regarding seizure management, particularly in the home setting. The Delphi method is frequently used to develop clinical recommendations in areas characterised by limited or heterogeneous evidence [20,22].

#### 2.6.1. The Delphi Study Design

A modified Delphi design was used, characterised by:

- the use of a predefined set of items, derived from:
  ○ the results of the current observational study
  ○ relevant international guidelines
  ○ recent literature (2019–2025)
- the conduct of two rounds of evaluation
- the use of a 9-point Likert scale to quantify the degree of agreement

This approach is specific to modified Delphi studies aimed at validating clinical recommendations and is considered appropriate when a preliminary evidence base already exists [16,18].

#### 2.6.2. Expert Panel

The Delphi panel comprised 8 experts with relevant experience in: paediatric neurology; paediatric palliative care; general paediatrics; specialist medical care (nurses)

The inclusion criteria were: a minimum of 5 years’ clinical experience; direct involvement in the care of paediatric patients; experience in the management of seizures.

The selection of the panel aimed to ensure a multidisciplinary perspective and to reflect real-world clinical practice, in line with recommendations for Delphi studies in the health sector [21,22].

Panel members completed all evaluations independently and had no access to the individual responses of other participants.

#### 2.6.3. Development of Consensus Items

The statements under review were developed on the basis of: the results of the observational study; international guidelines; and recent specialist literature.

The items were organised into ten clinical domains: assessment, risk stratification, maintenance antiepileptic treatment, management of acute seizures, treatment escalation, status epilepticus, home management, advance care planning, end-of-life care and organisational aspects of care.

For operational purposes, the final consensus recommendations were subsequently organised into ten domains within Appendix A, including both clinical and organisational aspects of seizure management in paediatric palliative care.

The formulation of the items adhered to the principles of clarity, clinical relevance and practical applicability, in accordance with the Delphi methodologies used in the development of the guidelines [21].

Statements that did not reach the predefined consensus threshold were reviewed for clarity, wording and clinical applicability based on aggregated expert feedback before being re-evaluated during the second Delphi round.

#### 2.6.4. Consensus Process

In the first round, the experts assessed each statement using a 9-point Likert scale:

- 1–3: disagreement
- 4–6: uncertainty
- 7–9: agreement

In the second round, statements that had not reached consensus were revised based on aggregated feedback and re-evaluated.

The definition of consensus was established a priori:

- consensus: ≥75% scores of 7–9
- strong consensus: ≥85% scores of 7–9

The use of these thresholds is frequently reported in clinical Delphi studies and allows for a standardised interpretation of expert agreement [22,23].

All evaluations were performed independently by panel members, and responses were collected anonymously using electronic questionnaires.

#### 2.6.5. Integration of Results

The results of the Delphi process were used to develop a structured clinical protocol for the management of seizures in paediatric palliative care, presented as Appendix A.

This protocol aims to: standardise the clinical approach; facilitate the management of acute seizures; support care in both hospital and home settings; and reduce variability in clinical practice.

Integrating expert consensus with existing observational data is recommended when developing clinical interventions in areas with limited evidence [20,21].

## 3. Results

### 3.1. Patient Characteristics

During the study period, a total of 101 paediatric patients with life-limiting illnesses were included in the study; their demographic and clinical characteristics are presented in Table 1.

The mean age of the patients was 7.2 ± 4.7 years, indicating a wide distribution of age groups within the cohort analysed. In terms of gender distribution, a slight predominance of males was observed (54.5%). The majority of patients were from rural areas (75.2%), a finding that reflects the differential access to specialist medical services and the profile of the population served by the palliative care service.

Regarding the primary diagnosis, neurological diseases were the dominant category, present in 74.3% of patients. The remaining cases included other categories of life-limiting diseases, with no specific aetiology predominating.

The assessment of neurological status revealed a high degree of impairment within the study population. Thus, structural brain lesions were identified in 92.1% of patients, severe neurodevelopmental delay in 86.1%, and severe motor impairment in 70.3%. These data indicate an increased prevalence of complex neurological impairment in the cohort analysed.

The clinical profile identified suggests the need for continuous multidisciplinary interventions, given the frequent association between severe neurological disability, functional dependence and the increased need for medical support.

Additional review of the clinical documentation indicated that many patients required complex supportive interventions, including enteral feeding and respiratory assistance, reflecting the advanced stage of neurological and functional impairment within the cohort. EEG data were available only for a subset of patients because investigations had been performed according to individual clinical indications rather than through a standardised protocol. Among the available reports, the most frequently described abnormalities included diffuse background slowing, multifocal epileptiform activity and severe encephalopathic patterns. These findings are consistent with the severe neurological phenotypes commonly described in children with developmental and epileptic encephalopathies receiving palliative care [10,11,13].

No standardised data regarding seizure frequency, epilepsy syndromes or drug-resistant epilepsy were consistently available in the retrospective records; therefore, these variables could not be analysed systematically. Consequently, the present results should be interpreted primarily as a descriptive characterisation of epilepsy within a clinically heterogeneous paediatric palliative care population.

The data summarised in Table 1 highlight that the study population is characterised by a predominance of neurological pathology and a high level of clinical severity, reflected in the increased frequency of structural brain damage and associated neuromotor disabilities.

### 3.2. Prevalence of Epilepsy

Epilepsy was documented in 33 of the 101 patients included in the study, corresponding to a prevalence of 32.7%.

The distribution of this condition within the cohort indicates that approximately one-third of children in palliative care present with epileptic manifestations in the context of their underlying disease. The presence of epilepsy was recorded based on clinical data in the medical records and associated anticonvulsant treatment.

The prevalence observed in the current cohort supports the hypothesis that epilepsy is one of the main neurological manifestations associated with life-limiting paediatric diseases, particularly in the presence of structural brain damage.

### 3.3. Comparative Analysis Between Patients with and Without Epilepsy

To assess possible associations between epilepsy and the clinical characteristics of patients, a comparative analysis was conducted between the group of patients with epilepsy (*n* = 33) and those without epilepsy (*n* = 68), with the results presented in Table 2.

Patients with epilepsy had a significantly higher prevalence of neurological disorders compared with those without epilepsy (90.9% vs. 66.2%; *p* = 0.008). Furthermore, severe neurodevelopmental delay was more common in the epilepsy group (97.0% vs. 80.9%; *p* = 0.032). Similarly, severe motor impairment was reported in a higher proportion of patients with epilepsy (84.8% vs. 63.2%; *p* = 0.036).

Comparison of the two groups reveals a statistically significant association between the presence of epilepsy and the severity of neurological impairment. The variables analysed (neurological diagnosis, neurodevelopmental delay and motor deficit) are more common in the group of patients with epilepsy, suggesting a correlation between epilepsy and the neurological complexity of patients.

### 3.4. Clinical Complexity Indicators

Clinical complexity indicators were defined as markers of severe neurological and functional impairment associated with increased care dependency and multidisciplinary support needs in paediatric palliative care populations. These indicators included structural brain damage, severe neurodevelopmental delay, severe motor impairment and the requirement for supportive interventions such as enteral feeding or respiratory assistance [5,13].

The overall clinical complexity indicators for the study population are presented in Table 3.

Structural brain damage was present in 92.1% of patients, severe neurodevelopmental delay in 86.1%, and severe motor impairment in 70.3%. Furthermore, a predominance of neurological comorbidity was observed within the cohort.

The data reflect a high level of clinical complexity among the included patients, characterised by the frequent association of several types of severe neurological impairment. This structure of the study population supports the multidimensional nature of paediatric palliative care and the need for an integrated approach.

These observational results formed the basis for the development of the statements subsequently evaluated in the Delphi process.

### 3.5. Results of the Delphi Process

The consensus process, conducted using a modified Delphi method, involved two rounds of evaluation of the clinical statements by the expert panel.

All evaluations were performed independently by panel members. Responses were collected anonymously using electronic questionnaires. After the first round, participants received aggregated feedback summarising the distribution of scores and comments. Statements that did not achieve the predefined consensus threshold were revised for clarity and re-evaluated during the second round.

In the first round, a total of 13 statements were evaluated, formulated based on the results of the observational study, international guidelines and recent literature. Most items reached the consensus threshold in the first round, whilst statements without consensus were revised and re-evaluated in the second round.

Following the completion of the two Delphi rounds, all 13 statements achieved strong consensus.

The distribution of agreement among experts highlighted a high level of convergence for areas considered essential in seizure management, including neurological assessment, risk stratification, acute seizure management and advanced care planning.

The highest levels of consensus were observed for:

- the need for pharmacological intervention in prolonged seizures
- the use of benzodiazepines as first-line treatment
- training families in the management of seizures at home

Detailed results of the consensus process, including scores for each statement and the distribution of responses, are presented in Appendix A.

A summary of the level of agreement for each statement is presented in Table 4.

**Figure 1:**
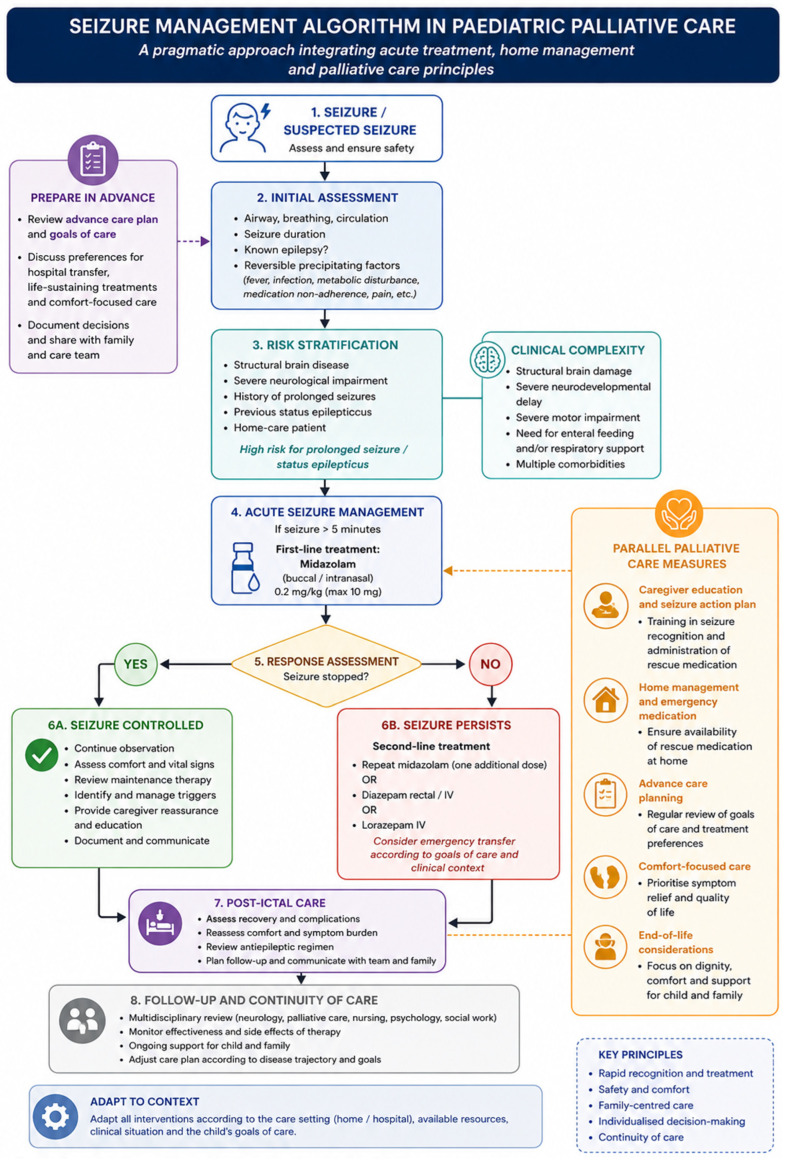
Pragmatic seizure management algorithm adapted for paediatric palliative care, integrating acute seizure treatment, home-based management, advanced care planning and comfort-oriented care principles.

Based on the results of the observational study and the consensus reached using the Delphi method, a clinical algorithm for the management of seizures in paediatric palliative care was developed, as shown in Figure 1.

## 4. Discussion

The results of this study highlight a significant prevalence of epilepsy (32.7%) among children in paediatric palliative care, confirming that neurological manifestations represent a major component of the clinical burden in this population. The analysis conducted in this study indicates that epilepsy is frequently associated with severe neurological impairment and functional dependence in children receiving paediatric palliative care. This finding is consistent with recent international literature, where the prevalence of epilepsy among children with life-limiting illnesses ranges from 20% to 50%, depending on the composition of the cohorts analysed and the prevalence of neurological pathology [7,10,33].

A key finding of the study is the significant association between epilepsy and severe neurological impairment. This observation is supported by recent research demonstrating that epilepsy frequently occurs in the context of structural encephalopathies and genetic disorders, and is closely correlated with the severity of neurodevelopmental impairment [10,11,28].

In particular, studies published since 2020 highlight the fact that epilepsy associated with severe neurological disorders is frequently drug-resistant and is associated with profound motor and cognitive disability [11,12]. The data obtained from the cohort analysed, in which almost all patients with epilepsy presented with severe neurodevelopmental delay (97.0%) and significant motor impairment (84.8%), are consistent with these observations.

Furthermore, the high proportion of structural brain abnormalities (92.1%) in the study population supports the hypothesis that epilepsy is closely linked to the neuropathological substrate. Recent studies show that structural brain changes constitute one of the main determinants of epileptogenesis in children, particularly in the context of neurodegenerative or genetic disorders [5,12].

The results of the comparative analysis indicate that epilepsy is significantly associated with indicators of clinical severity, such as neurological diagnosis, neurodevelopmental delay and motor deficit. These findings suggest that epilepsy is frequently associated with severe neurological and functional impairment in paediatric palliative care populations.

Recent literature highlights that paediatric patients with epilepsy associated with life-limiting conditions have a higher symptom burden and require a greater level of medical care and interdisciplinary support [33,34]. Furthermore, recurrent seizures may contribute to additional neurological burden and functional deterioration in vulnerable patients with severe underlying neurological disorders [11,12].

In addition to its biological impact, epilepsy also has a significant psychosocial dimension. Seizures are perceived as acute, unpredictable and potentially threatening events, which generate increased levels of stress and anxiety among families. Recent studies have shown that the presence of seizures is associated with a significant decline in quality of life for both the patient and carers [5,34].

From a clinical perspective, the results of this study support the need for a proactive approach to the management of epilepsy in paediatric palliative care. Current literature recommends the integration of systematic neurological assessment and the use of individualised therapeutic strategies, adapted to the palliative care context [13,14,35].

In this context, the observational findings were used as the basis for developing a clinical protocol for seizure management through a consensus-based approach using the modified Delphi method. The integration of empirical data with clinical expertise is recommended in fields characterised by limited evidence, such as paediatric palliative care, where randomised trials are difficult to conduct [20,22].

The Delphi process revealed a high level of consensus among experts, with all statements evaluated meeting the established consensus threshold and the majority achieving a strong level of consensus. This degree of convergence suggests the existence of relatively well-established clinical practices, even in the absence of formalised protocols, and supports the feasibility of developing standardised recommendations.

The proposed algorithm was not intended to replace existing international epilepsy guidelines, such as NICE or ILAE recommendations, but rather to adapt general seizure management principles to the practical realities of paediatric palliative care, particularly in home-care settings and resource-limited environments. Unlike standard epilepsy management pathways, the algorithm incorporates elements specific to paediatric palliative care, including advance care planning, caregiver training, home-based emergency management and comfort-oriented decision-making. The primary objective was to provide a pragmatic framework capable of supporting symptom-oriented management and continuity of care in clinically complex patients.

The proposed algorithm should therefore be regarded as a consensus-based framework intended to support clinical decision-making rather than a validated intervention, and prospective studies are needed to evaluate its impact on clinical outcomes.

The areas that achieved the highest level of consensus include the management of acute seizures, the use of benzodiazepines as first-line treatment, and the need for training families in home-based intervention. These results are consistent with international guidelines, which recommend the use of alternative routes of administration for rapid seizure control and the prevention of progression to status epilepticus [36,37].

A particularly relevant aspect is the emphasis placed by experts on the management of seizures in the home setting and on advance care planning. In the context of paediatric palliative care, where most interventions take place outside the hospital setting, these elements are essential for ensuring continuity of care and reducing emergency interventions [34,37].

In recent years, new treatment options for epilepsy have been developed, including drugs with innovative mechanisms and adjuvant therapies. However, in the paediatric palliative care population, complete seizure control is often difficult to achieve, and the primary objective remains reducing the frequency and severity of episodes [12,13].

A key point highlighted by recent literature is the importance of advanced plans for seizure management, particularly in the home setting. The rapid administration of benzodiazepines via alternative routes has proven effective in preventing progression to status epilepticus and in reducing the need for emergency interventions [35,37]. Furthermore, training families and carers is a critical component of care.

Studies show that educating them contributes to increased adherence to treatment and improved management of acute episodes [5,37].

A relevant aspect of the study is the high proportion of patients from rural areas (75.2%), which reflects the specific socio-economic context of Romania and the Eastern European region.

Global data indicate that access to diagnosis and treatment for epilepsy is unevenly distributed, with a significant impact in resource-limited countries [38]. In this context, palliative care services play a vital role in reducing these disparities by providing continuous care and multidisciplinary support.

### Limitations of the Study

The results of this study should be interpreted in light of several methodological limitations. First, the retrospective observational design relied on information available in medical records, which may have introduced reporting bias and variability in data quality. Second, the study population was clinically heterogeneous, including patients with different neurological, genetic, metabolic and other life-limiting conditions, which may limit the comparability of findings across diagnostic categories.

Furthermore, detailed epilepsy-related variables, including seizure frequency, epilepsy syndromes, drug-resistant epilepsy status and longitudinal treatment response, were not consistently documented and therefore could not be analysed systematically. EEG and laboratory investigations were also not uniformly available because neurological monitoring was performed according to individual clinical indications and resource availability rather than through a standardised protocol.

The statistical analysis was primarily exploratory and descriptive in nature. Due to the relatively limited sample size and the structure of the available data, multivariate analyses adjusting for potential confounding variables were not performed. Consequently, the reported associations should not be interpreted as independent predictors or causal relationships.

In addition, the Delphi component involved a relatively small expert panel and several recommendations reflected established clinical practices already described in international guidelines. The proposed protocol has not yet undergone prospective validation, and its applicability outside the studied setting remains uncertain. Finally, the single-centre design may further limit the generalisability of the findings to other paediatric palliative care systems or healthcare environments [21,22,23].

Consequently, the algorithm should be interpreted as a pragmatic consensus-based tool rather than evidence of improved clinical outcomes.

## 5. Conclusions

This study provides a descriptive overview of epilepsy among children receiving paediatric palliative care in a clinically complex population from an Eastern European setting. The findings indicate frequent associations between epilepsy and severe neurological impairment, highlighting the importance of systematic neurological assessment and symptom-oriented management strategies in this context.

The integration of observational clinical data with expert consensus enabled the development of a pragmatic seizure management protocol adapted to paediatric palliative care practice, particularly for home-based care and resource-limited settings. Rather than replacing existing international recommendations, the proposed framework aims to facilitate continuity of care and support clinically appropriate decision-making in complex cases.

Although exploratory and descriptive in nature, the study contributes additional clinical data from a region where paediatric palliative care research remains limited. Further prospective multicentre studies are required to validate the proposed protocol and to better characterise epilepsy-related outcomes in paediatric palliative care populations.

## Figures and Tables

**Table 1:** Patient characteristics (*n* = 101).

Variable	Value
Total number of patients	101
Age (years), mean ± SD	7.2 ± 4.7
Male, *n* (%)	55 (54.5%)
Female	46 (45.5%)
Rural, *n* (%)	76 (75.2%)
Neurological diseases, *n* (%)	75 (74.3%)
Structural brain injury, *n* (%)	93 (92.1%)
Severe neurodevelopmental delay, *n* (%)	87 (86.1%)
Severe motor impairment, *n* (%)	71 (70.3%)
Epilepsy, *n* (%)	33 (32.7%)

**Table 2:** Comparative analysis (epilepsy vs. no epilepsy).

Variable	Epilepsy (*n* = 33)	No Epilepsy (*n* = 68)	*p*-Value
Neurological diseases, *n* (%)	30 (90.9%)	45 (66.2%)	0.008
Severe neurodevelopmental delay, *n* (%)	32 (97.0%)	55 (80.9%)	0.032
Severe motor impairment, *n* (%)	28 (84.8%)	43 (63.2%)	0.036

**Table 3:** Clinical complexity indicators in the study population.

Indicator	*n* (%)
Structural brain damage	93 (92.1%)
Severe delay	87 (86.1%)
Severe motor impairment	71 (70.3%)

**Table 4:** Results of the Delphi process (summary).

No.	Statement (Summary)	Agreement (%)	Level of Consensus
1	Systematic neurological assessment in all patients	94%	High consensus
2	Identification of patients at high risk of seizures	91%	High consensus
3	Maintenance and adjustment of antiepileptic treatment	88%	Substantial agreement
4	Pharmacological intervention for seizures lasting ≥5 min	96%	Strong consensus
5	Use of oral/intranasal midazolam as first-line treatment	92%	High consensus
6	Escalation of treatment following benzodiazepine failure	89%	High level of agreement
7	Treatment of prolonged seizures as a medical emergency	100%	Strong consensus
8	Training families in seizure management	95%	High consensus
9	Availability of emergency medication at home	97%	High consensus
10	Development of individualised advance care plans	93%	Strong consensus
11	Prioritising comfort in end-of-life care	98%	Strong consensus
12	Use of palliative sedation in refractory cases	86%	Strong consensus
13	The need to implement standardised protocols	90%	Strong consensus

## Data Availability

All data from the first author and the corresponding author are available on request.

## Appendix A

Consensus-based clinical recommendations for the management of seizures in paediatric palliative care: a modified Delphi study.

### Appendix A.1. Rationale and Objectives

Given the high prevalence of seizures among children in paediatric palliative care and the absence of standardised protocols tailored to this context, a consensus-based approach was used to develop applicable clinical recommendations.

The aim of this approach was to integrate the available evidence with the clinical experience of experts, in order to develop a practical framework for the management of seizures, including in the context of home care.

### Appendix A.2. Study Design

A modified Delphi study was conducted, a method frequently used for developing clinical recommendations in the absence of high-level evidence or in areas with high variability in practice [20,21].

### Appendix A.3. Composition of the Expert Panel

The expert panel comprised 8 specialists with relevant experience in: paediatric neurology; paediatric palliative care; general paediatrics; specialist nursing (nurses).

Inclusion criteria: a minimum of 5 years’ clinical experience; direct involvement in the care of paediatric patients; experience in the management of seizures.

### Appendix A.4. Development of Consensus Items

The initial set of statements was developed based on:

- the results of the present observational study;
- international guidelines [17,36,39]
- recent scientific literature (2019–2025).

The statements were grouped into the following domains:

1. Assessment
2. Risk stratification
3. Mainstream treatment
4. Management of acute seizures
5. Escalation of treatment
6. Management at home
7. End-of-life care

### Appendix A.5. The Delphi Process

Round 1

The experts rated each statement using a 9-point Likert scale:

- 1–3: disagree
- 4–6: uncertainty
- 7–9: agreement

Round 2

Statements without consensus were reviewed and re-evaluated.

Definition of consensus

- consensus: ≥75% scores of 7–9
- strong consensus: ≥85% scores of 7–9

### Appendix A.6. Results of the Consensus Process

Following two Delphi rounds, 13 clinical recommendations were validated, of which 10 achieved a high level of agreement (>85%).

### Appendix A.7. Consensus-Based Recommendations

Domain 1: Assessment

Statement 1

A systematic neurological assessment should be carried out in all patients receiving paediatric palliative care.

Agreement: 94% (strong consensus)

Domain 2: Risk stratification

Statement 2

Patients with structural brain damage, known epilepsy or severe disability should be considered at high risk of seizures.

Agreement: 91% (strong consensus)

Domain 3: Maintenance antiepileptic treatment

Statement 3

Antiepileptic treatment should be maintained and adapted to the patient’s clinical condition.

Agreement: 88% (strong consensus)

Domain 4: Management of acute seizures

Statement 4

Seizures lasting ≥5 min require pharmacological intervention.

Agreement: 96% (strong consensus)

Statement 5

Oral or intranasal midazolam should be used as first-line treatment.

Agreement: 92% (strong consensus)

Domain 5: Treatment escalation

Statement 6

In the absence of a response to benzodiazepines, second-line treatment should be initiated.

Agreement: 89% (strong consensus)

Domain 6: Status epilepticus

Statement 7

Prolonged or refractory seizures should be treated as medical emergencies.

Agreement: 100% (strong consensus)

Domain 7: Home management

Statement 8

Families should be trained in the recognition and management of seizures.

Agreement: 95% (strong consensus)

Statement 9

Emergency medication must be available at home.

Agreement: 97% (strong consensus)

Domain 8: Advance care planning

Statement 10

High-risk patients should have personalised advance care plans.

Agreement: 93% (strong consensus)

Domain 9: End-of-life care

Statement 11

Seizure management should prioritise the patient’s comfort.

Agreement: 98% (strong consensus)

Statement 12

Palliative sedation may be used in refractory cases.

Agreement: 86% (strong consesnus)

Domain 10: Organisational level

Statement 13

The implementation of standardised protocols is essential in paediatric palliative care.

Agreement: 90% (strong consensus)

### Appendix A.8. Integration into Clinical Practice

The recommendations obtained have been integrated into a structured clinical protocol, with the aim of facilitating their application in daily practice.

### Appendix A.9. Strengths and Limitations

Strengths:

- integration of real-world clinical data with expert opinion;
- a structured and reproducible approach;
- high practical applicability.

Limitations:

- the size of the panel;
- possible regional bias;
- lack of prospective validation.

### Appendix A.10. Future Directions

- prospective validation of the protocol;
- assessment of the impact on quality of life;
- adaptation for resource-limited settings.
